# Uncovering steroidopathy in women with autism: a latent class analysis

**DOI:** 10.1186/2040-2392-5-27

**Published:** 2014-04-09

**Authors:** Alexa Pohl, Sarah Cassidy, Bonnie Auyeung, Simon Baron-Cohen

**Affiliations:** 1 Autism Research Centre, Department of Psychiatry, University of Cambridge, Douglas House, 18B Trumpington Road, Cambridge CB2 8AH, UK; 2 Department of Psychology and Behavioural Sciences, Coventry University, James Starley Building, Cox Street, Coventry CV1 5LW, UK; 3 Department of Psychology, University of Edinburgh, 7 George Square, Edinburgh EH8 9 AD, UK; 4 CLASS Clinic, Cambridgeshire and Peterborough Mental Health Foundation NHS Trust, The Chitra Sethia Autism Centre, The Gatehouse, Fulborn Hospital, Fulborn, Cambridge CB21 5EF, UK

**Keywords:** Autism, Sex steroids, Polycystic ovary syndrome, Testosterone, Hormones

## Abstract

**Background:**

Prenatal exposure to increased androgens has been implicated in both polycystic ovary syndrome (PCOS) and autism spectrum conditions (ASC), suggesting that PCOS may be increased among women with ASC. One study suggested elevated steroidopathic symptoms (‘steroidopathy’) in women with ASC. As the symptoms are not independent, we conducted a latent class analysis (LCA). The objectives of the current study are: (1) to test if these findings replicate in a larger sample; and (2) to use LCA to uncover affected clusters of women with ASC.

**Methods:**

We tested two groups of women, screened using the Autism Spectrum Quotient - Group 1: n = 415 women with ASC (mean age 36.39 ± 11.98 years); and Group 2: n = 415 controls (mean age 39.96 ± 11.92 years). All participants completed the Testosterone-related Medical Questionnaire online. A multiple-group LCA was used to identify differences in latent class structure between women with ASC and controls.

**Results:**

There were significant differences in frequency of steroid-related conditions and symptoms between women with ASC and controls. A two-class semi-constrained model best fit the data. Based on response patterns, we identified the classes as ‘Typical’ and ‘Steroidopathic’. The prevalence of the ‘Steroidopathic’ class was significantly increased within the ASC group (Δ*G*^2^ = 15, *df* =1, *P* = 0.0001). In particular, we confirmed higher frequencies of epilepsy, amenorrhea, dysmenorrhea, severe acne, gender dysphoria, and transsexualism, and differences in sexual preference in women with ASC.

**Conclusions:**

Women with ASC are at increased risk for symptoms and conditions linked to steroids. LCA revealed this steroidopathy despite the apparent underdiagnosis of PCOS.

## Background

Atypical levels of sex steroid hormones and their biosynthetic pathway have been associated with autism spectrum conditions (ASC) or associated with autistic traits in genetic [1-3], gene expression [4,5], serum [6,7], amniotic fluid [8] and 2D:4D ratio of 2nd digit to 4th digit [9] studies. Elevated levels of prenatal androgens such as fetal testosterone (FT) during a critical period are hypothesized to contribute to the etiology of ASC [10] as FT shapes neurological development [11-13]. Prenatal androgens also contribute to the programming of the hypothalamic-pituitary-gonadal (HPG) axis [14,15], and elevated levels of prenatal androgens also contribute to the etiology of polycystic ovary syndrome (PCOS) [16]. The present study further explores the link between ASC and PCOS.

Many candidate genes implicated in ASC are also implicated in female reproductive disorders. For example, a shorter CAG variable number tandem repeat associated with hypersensitivity of the androgen receptor [17] has been linked to ASC [3], precocious puberty, and PCOS [18]. *CYP19A1*, whose product aromatase converts testosterone to estradiol and androstenedione to estrone, has been related to ASC [1], PCOS [19,20], and female hyperandrogenism [21]. Additionally, *CYP19A1* has been identified as a target of transcriptional regulators such as *RORA*, which is associated with autism [2], autoimmunity [22] and early age at menarche [23]. Another gene of interest in both autism [1] and female reproductive disorders [24] is *CYP17A1*, whose product 17α-hydroxylase/17,20 lysase/17,20-desmolase catalyzes several steroidogenic reactions. *CYP17A1* is overexpressed in theca cells of women with PCOS [25], and its overexpression is driven by increased insulin receptor signaling, [26] such that hyperinsulinemia can eventually cause hyperandrogenemia [27]. Autism is associated with *CYP11B1*, which encodes 11β-hydroxysteroid dehydrogenase type 1 (11β-HSD-1) [1]. Inactivating mutations in 11β-HSD-1 cause androgenic precursors to accumulate in the adrenal gland, resulting in congenital adrenal hyperplasia (CAH), a condition that can virilize females *in utero*[28]. Consistent with the hypothesized programming effects of prenatal androgens, females with CAH score significantly higher than their unaffected sisters on the Autism Spectrum Quotient (AQ), a self-report measure of autistic traits [29].

A few studies have implicated hyperandrogenemia in ASC. However, it is not clear whether the rise in androgens is mediated by the gonads or the adrenals, or is found in both sexes and across the whole of the lifespan. Testosterone, luteinizing hormone (LH) [7] and androstenedione [6] are all elevated in women with ASC, but of these only androstenedione is also elevated in men with ASC [6,7]. DHEA-S, DHEA [30] and testosterone [31] are elevated in pre-pubertal or pubertal ASC males, but are decreased or unchanged relative to controls in adolescent and adult ASC males [6,32-34].

Animal models of prenatal androgenization (PNA) consistently produce a hyperandrogenic, anovulatory, insulin-resistant, viscerally obese phenotype [16], with epigenetic changes in genes involved in ovarian folliculogenesis, insulin signaling, and the HPG axis [35]. However, the effects of PNA on the timing of puberty remain unclear. For example, rhesus macaques with PNA exhibit delayed puberty, but mice with PNA exhibit early puberty [36]; additionally, pubertal androgen levels and nutrition affect the timing of menarche as well [37]. Similarly, PCOS is associated with both early puberty [38,39] and delayed menarche [40].

While hyperandrogenemia is correlated with a set of reproductive symptoms, testosterone is only one of a series of inter-related metabolites that includes the primary ovarian hormones progesterone and estradiol. Consequently, variation in the steroidogenic pathway in females with ASC would be expected to also result in symptoms or conditions related to abnormal levels of progesterone and/or estradiol, such as endometriosis [41] or steroid-sensitive cancers. Additionally, women with ASC would be expected to have increased susceptibility to psychiatric and neurological conditions mediated by gonadal hormones, such as premenstrual dysphoric disorder (PMDD) [42], anorexia [43], or epilepsy [44].

Previously, we reported significant increases in the frequency of steroid hormone-associated and sex-linked conditions in women with ASC and in mothers of children with ASC [45]. In the current study, we (1) test if these findings replicate in a larger sample, and (2) examine the patterns of reported reproductive symptoms and conditions to understand if differences exist between subgroups of women with and without ASC.

## Methods

### Participants

We recruited two groups of participants from the Cambridge Autism Research Centre Database, which consists of volunteers registered either at the Autism Research Centre (http://www.autismresearchcentre.com) or Cambridge Psychology (http://www.cambridgepsychology.com) volunteer websites. As a check on the difference between the groups, we administered the AQ [46].

#### Women with an autism spectrum condition

In total, 415 women with an ASC participated in the survey. These comprised 260 women with Asperger Syndrome, 27 with high functioning autism, and 128 women with an ASC that was not further specified. The mean (±SD) age of the ASC group was 36.39 ± 11.98 years. The mean AQ (±SD) of the respondents with ASC who also completed the AQ (n = 379) was 35.54 ± 10.96, in line with previous studies [46]. Additionally, participants were asked for the qualification of the individual who diagnosed them (for example, psychiatrist, clinical psychologist), the clinic where the diagnosis was made, and the date of their diagnosis.

#### Female controls

In total, 415 women without an ASC participated in the study, after exclusions. We excluded women with an AQ greater than 1 SD (AQ ≥23) above the population mean from previously reported samples, following established methods in previous studies [47], to ensure undiagnosed cases of ASC were excluded from the control group. We also excluded participants who reported having a relative with an ASC to minimize the likelihood that the control group might have a Broader Autism Phenotype [47]. The control participants had a mean age of 39.96 ± 11.92 years.

### Ethics

The Cambridge Autism Research Centre Database received ethical approval from the Psychology Research Ethics Committee at the University of Cambridge.

### Measures

All participants took the Testosterone-related Medical Questionnaire (TMQ), which has previously been used [45] (see Additional file 1: Supplementary Appendix 1). The TMQ was completed online. We assumed consent on the return of a completed questionnaire.

### Analysis

We computed frequency tables for women with ASC versus control women for each item on the TMQ. Previous research has shown that rates of contraceptive pill (CP) use are lower among women with an ASC [45]. Additionally, the CP can reduce many of the symptoms on the TMQ. In order to ensure that CP use was not affecting symptoms reported by participants, we used log-linear analysis to check for three-way interactions between group, contraceptive use, and reproductive or sex steroid-related items (PCOS, premenstrual syndrome (PMS), excessive menstrual bleeding, hirsutism, amenorrhea (irregular menstrual cycle), dysmenorrhea (unusually painful periods) and severe acne) and two-way interactions between contraceptive use and each symptom. Only parous women were considered on obstetric items (miscarriage, pre-eclampsia, and difficulty conceiving). Unadjusted odds ratios were calculated from each two-by-two frequency table. Fisher’s one-sided exact test was used to calculate significance, unless otherwise noted. SPSS was used to calculate all frequency-based statistics SPSS Statistics version 21, IBM, IBM Corporation, 1 New Orchard Road,Armonk, New York 10504-1722, United States.

As the questionnaire was designed to measure endocrine symptoms and conditions, we surmise that response patterns on the questionnaire reflect underlying endocrine status. For example, the likelihood of a participant reporting amenorrhea is linked to her likelihood of reporting hirsutism, as both symptoms are associated with an increased free androgen index [48]. Latent class analysis (LCA) assumes that an individual’s responses on a series of observed variables can be explained by an individual’s status on an unmeasured, or latent, categorical variable. LCA identifies high frequency response patterns on the series of observed variables, and these response patterns are used to draw conclusions about the discrete subpopulations, or latent classes, within the study population. By using LCA, we can explore group differences across a profile of steroid-related symptoms or conditions.

LCA is often described as a categorical cousin of factor analysis. However, unlike factor analysis, it maintains a “person-centric”, rather than a “variable-centric”, approach. For each subpopulation identified, LCA returns item-specific conditional probabilities describing the likelihood that an individual would respond positively to specific items given membership in a given latent subpopulation. These conditional probabilities are used to characterize the subpopulations. LCA uses an expectation-maximization algorithm to determine the prevalence of latent classes within the population and the posterior probabilities describing class membership. By applying LCA to our data, we aimed to uncover interpretable clusters of individuals with similar steroid-related symptoms, and the prevalence of these clusters in women with and without autism.

Variables that may influence class membership can be entered as covariates in LCA. We entered body mass index (BMI) and contraceptive use as covariates, as both adiposity [49] and contraceptive use affect hormonal levels. Including BMI as a covariate reduced our sample size slightly to 342 participants with ASC, and 402 control participants. The Lo-Mendell-Rubin (LMR) test was used to compare models with *k* and *k-1* latent classes, and the Akaike Information Criterion (AIC) and Bayesian Information Criterion (BIC) were used to compare model fit (Additional file 2). We used multiple-group LCA to compare women with ASC to controls, allowing us to detect both qualitative differences (that is, number of subpopulations, conditional item-response probabilities for each subpopulation) and quantitative differences (that is, differences in subpopulation prevalence) between groups. Likelihood ratio tests (LRTs) were used to detect significant improvements in model fit as a result of including ‘ASC diagnosis’ as a grouping variable. All latent class modeling was done with MPlus Version 7 Muthén & Muthén, 3463 Stoner Avenue, Los Angeles, CA 90066.

## Results

Frequency differences on each item are shown in Table 1. Prior to assessing frequency differences between the groups, we used log-linear analysis to ascertain if contraceptive use was associated with any of the conditions and ASC diagnosis. There were no three-way interactions between ASC diagnosis, contraceptive use, and any steroid-related symptom or diagnosis, but we found significant interactions between contraceptive use and PCOS, PMS, menorrhagia, and severe acne. Subsequently, we report rates of PCOS, PMS, menorrhagia, and severe acne with respect to contraceptive use.

**Table 1:** Frequencies of reproductive and sex-linked symptoms and conditions among women with autism spectrum conditions and controls

**Condition or symptom**	**Women with ASC (n = 415)**	**Controls (n = 415)**	**Unadjusted odds ratio (95% CI)**	***P *** **value (Fisher's exact test, one-sided)**
	***(sample size, if different from above)***	***(sample size, if different from above)***		
	**n (%)**	**n (%)**		
**Sex-linked conditions**				
Anorexia	33 (8.0%)	10 (2.4%)	3.50 (1.70-7.20)	**0.000**
Congenital adrenal hyperplasia	1 (0.2%)	0 (0.0%)	-	0.500
Diabetes	10 (2.4%)	14 (3.4%)	0.71 (0.31-1.61)	0.268
Epilepsy	17 (4.1%)	6 (1.4%)	2.91 (1.14-7.46)	**0.016**
Any hormonal medical condition	43 (10.4%)	35 (8.4%)	1.23 (0.79-2.01)	0.203
Cardiac condition	16 (3.9%)	5 (1.2%)	3.29 (1.19-9.06)	**0.012**
Thyroid condition	35 (8.4%)	35 (8.4%)	1.00 (0.61-1.63)	0.550
Penicillin allergy	38 (9.2%)	37 (8.9%)	1.03 (0.64-1.66)	0.500
**Reproductive or sex steroid-related symptoms or conditions**				
Hirsutism	76 (18.3%)	61 (14.7%)	1.30 (0.90-1.88)	0.089
Irregular menstrual cycle	192 (46.3%)	141 (34.0%)	1.673 (1.26-2.21)	**0.0002**
Unusually painful periods	163 (39.3%)	109 (26.3%)	1.82 (1.35-2.44)	**0.00004**
Polycystic ovary syndrome				
Contraceptive pill users	33 (11.8%)	47 (14.4%)	0.80 (0.50-1.29)	0.212
	*279*	*327*		
Contraceptive pill non-users	11 (10.2%)	4 (4.7%)	2.296 (0.70-7.49)	0.126
	*108*	*85*		
Pre-menstrual syndrome				
Contraceptive pill users	67 (24.0%)	45 (13.8%)	1.98 (1.30-3.01)	**0.001**
	*279*	*327*		
Contraceptive pill non-users	12 (11.1%)	7 (8.2%)	1.39 (0.52-3.71)	0.339
	*108*	*85*		
Excessive menstrual bleeding				
Contraceptive pill users	94 (33.7%)	84 (25.7%)	1.47 (1.04-2.09)	**0.019**
	*279*	*327*		
Non-contraceptive pill users	22 (20.4%)	14 (16.5%)	1.30 (0.62-2.72)	0.308
	*108*	*85*		
Severe acne				
Contraceptive pill users	49 (17.6%)	41 (12.5%)	1.49 (0.95-2.33)	0.053
	*279*	*327*		
Non-contraceptive pill users	23 (21.3%)	5 (5.9%)	4.33 (1.57-11.94)	**0.002**
	*108*	*85*		
**Timing of puberty**				
Delayed puberty	4 (1.0%)	2 (0.5%)	2.01 (0.37-11.03)	0.343
Precocious puberty	13 (3.1%)	2 (0.5%)	6.68 (1.50-29.78)	**0.003**
Early growth spurt	84 (20.2%)	53 (12.8%)	1.73 (1.19-2.521)	**0.002**
Periods after 16 years	28 (6.7%)	19 (4.6%)	1.51 (0.83-2.75)	0.115
Periods before 10 years	34 (8.2%)	23 (5.5%)	1.52 (0.88-2.63)	0.085
**Reproductive cancers**				
Breast cancer	17 (4.1%)	15 (3.6%)	1.14 (0.56-2.31)	0.429
Family history of breast cancer	97 (23.4%)	106 (25.5%)	0.89 (0.65-1.22)	0.259
Ovarian cancer	9 (2.2%)	12 (2.9%)	0.74 (0.31-1.79)	0.330
Family history of ovarian cancer	49 (11.8%)	41 (9.9%)	1.22 (0.79-1.90)	0.217
Family history prostate cancer	39 (9.4%)	44 (10.6%)	0.88 (0.56-1.34)	0.322
Uterine cancer	17 (4.1%)	12 (2.9%)	1.43 (0.68-3.04)	0.225
Family history uterine cancer	43 (10.4%)	34 (8.2%)	1.30 (0.81-2.08)	0.169
**Gender and sexuality**				
Tomboyism	371	404		
	252 (67.9%)	171 (42.3%)	2.885	**0.0000**
Gender dysphoria	*320*	*396*		
	12 (3.8%)	1 (0.3%)	15.39	**0.0004**
Sexual preference				
	*414*	*415*		0.000*
Male	261 (63.0%)	369 (88.9%)		
Female	48 (11.6%)	28 (6.7%)		
Both	57 (13.8%)	18 (4.3%)		
Neither	48 (11.6%)	0 (0.0%)		
Transsexual	*102*	*52*		
	12 (11.8%)	1 (1.9%)	6.80 (0.86-53.82)	**0.030**
**Obstetric conditions**	ASC (parous)	Controls (parous)		
	*185*	*264*		
Miscarriage	56 (30.3%)	65 (24.6%)	1.33 (0.87-2.02)	0.111
Pre-eclampsia	12 (6.5%)	17 (6.4%)	1.01 (0.47-2.16)	0.565
Difficulty conceiving	25 (13.5%)	51 (19.3%)	0.65 (0.39-1.10)	0.068

*A chi-squared statistic was calculated instead of using Fisher’s exact test. *P* values shown in bold are significant (*P* < 0.05). ASC, autism spectrum conditions.

Significant differences between women with ASC and controls were found for self-reported rates of anorexia (*P* < 0.0001), epilepsy (*P* = 0.016), and cardiac conditions (excluding cardiac arrhythmia) (*P* = 0.012). Additionally, self-report rates of testosterone-related symptoms including irregular menstrual cycles (*P* = 0.0002) and unusually painful periods (*P* < 0.0001) were higher among women with ASCs than controls. Self-reported rates of hirsutism, a characteristic feature of PCOS, were higher among women with ASC, though the trend did not reach significance (*P* = 0.089). Among contraceptive users, women with ASC reported PMS (*P* = 0.001) and menorrhagia (*P* = 0.019) significantly more frequently than controls. However, among non-contraceptive users, significantly more women with an ASC than controls reported having severe acne (*P* = 0.002). There was also a near significant difference in acne (*P* = 0.053) between women with an ASC who used the CP and controls who used the CP. Rates of precocious puberty (*P* = 0.003), and early growth spurt (*P* = 0.002) were significantly different between women with ASC and controls, while there was a trend towards significance in menarche prior to 10 years of age (*P* = 0.085). No differences in rates of hormone-responsive cancers (ovarian, uterine, or breast) or family history of hormone-responsive cancers (ovarian, uterine, breast, or prostate) were found between participants with ASC and controls. Similarly, no differences in gestational complications were found between parous women with an ASC (n = 185) and parous controls (n = 264).

Questions regarding gender identity and sexual orientation were optional; fewer participants responded to items on ‘tomboyism’, gender identity disorder, and transsexualism. Significantly more women with an ASC (n = 371) than controls (n = 404) reported being a ‘tomboy’ during childhood (*P* < 0.0001). Different self-report frequencies of gender identity disorder (also gender dysphoria) (*P* = 0.0004; ASC n = 320, control n = 396), and transsexualism (*P* = 0.030; ASC n = 102, control n = 52) were found between women with ASC and controls. Finally, participant sexual orientation (chi-squared; *P* < 0.001) differed significantly between women with ASC (n = 414) and controls (n = 415), with more women with ASC reporting bisexualism or lesbianism than among controls.

### Latent class analysis results

**Figure 1:**
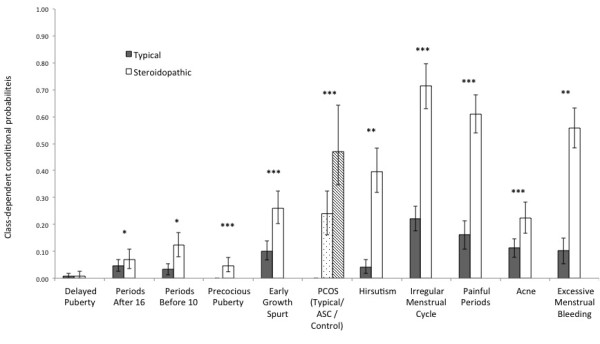
**Class-dependent conditional probabilities for Testosterone-related Medical Questionnaire items for steroidopathic and typical latent classes.** This figure demonstrates the strong separation between the latent classes. Error bars represent 95% confidence intervals generated over 500 bootstrap replications. For polycystic ovary syndrome (PCOS), the conditional probability is given for the ‘typical’ latent class, the ‘autism spectrum condition (ASC)-steroidopathic’ latent class, and for the ‘control-steroidopathic’ latent class, in order to demonstrate the between-group differences on responses to this item. *P < 0.05, **P < 0.01, ***P < 0.001, significant difference between classes.

Determining the correct number of latent classes is the first step in LCA. To chose the correct number of latent classes for our analysis, we minimized the AIC and BIC by choosing a model with *k* classes, and we used the LMR test to verify that the model with *k* classes fit better than the model with *k-1* classes (Table 2). The three-class model had the lowest AIC, but the two-class model had the lowest BIC. The BIC tends to outperform the AIC at identifying the best model when the sample size is large in LCA [50]. The LMR test supported the two-class model, as the three-class model did not fit better than the two-class model (*P* = 0.6174). Consequently, we determined that the two-class model was the best representative of the true latent class structure, and used a *k* = 2 model for our multigroup LCA. Clear divisions existed between the classes, with one class having low and the other class having high probabilities of reporting early growth spurt, PCOS, hirsutism, irregular menstrual cycles, painful periods, severe acne, and excessive menstrual bleeding (Figure 1).

**Table 2:** **Information criteria for models of *k *** **classes and Lo-Mendell-Rubin test for improvement between models with *k *** **and *k-1 *** **classes**

***k *** **classes**	***df***	**-log likelihood**	**AIC**	**BIC**	**LMR test**
					***P *** **value**
1	8	-6,424.359	12,878.718	12,949.54	
2	21	-2,906.206	5,862.413	**5,977.781**	**0.0000**
3	30	-2,869.814	**5,817.628**	5,997.602	0.6174

The lowest values on the information criteria indicate the best model (shown in bold), weighing absolute model fit and number of estimated parameters. A description of the two and three class models can be found in Additional file 1: Table S1. AIC, Akaike Information Criterion; BIC, Bayesian Information Criterion; *df*, degrees of freedom; LMR, Lo-Mendell-Rubin. *P* value in bold indicates significance *P* < 0.05).

Because the intensity of sex steroid-related symptoms can vary with adiposity and hormonal CP use, we included BMI and CP use as covariates in our model. BMI was significantly associated with membership in the latent class characterized by increased risk of steroid-related symptoms and conditions (which we deemed the ‘steroidopathic’ class) (β_BMI_ = 0.118; *P* < 0.001), but CP use was not (β_CP_ = 0.277; *P* = 0.264). Similar results were found for BMI and CP use in all models.

In order to ascertain whether the prevalence and characteristics of the latent classes varied by ASC diagnosis, we performed a two-class LCA comparing women with ASC to controls. First, we compared an unconstrained model where both the number of women in each latent class and the item-response patterns within the latent classes were allowed to vary by diagnosis, to a semi-constrained model where only the number of women in each latent class was allowed to vary by diagnosis. This tested if the characteristics of the latent classes were similar between groups; no significant improvement in model fit was detected by allowing the item-response patterns to vary by diagnosis (ΔG^2^ = 20.309, *df* = 22, *P* = 0.0625, information criteria, Table 3; likelihood ratio tests, Table 4). However, the trend towards significance prompted us to further explore item-response posterior probabilities between groups. Subsequently, a Wald parameter test revealed that the difference in the posterior probability of reporting PCOS was significant (chi-squared = 11.743, *df* = 2, *P* = 0.0028); no other posterior probabilities differed significantly between groups. Allowing the posterior probability of reporting PCOS to vary while other parameters were held equal between groups improved all information criteria (Table 3), and significantly improved model fit (LRT; ΔG^2^ = 6.673, *df* = 2, *P* = 0.0178) (Table 4).

**Table 3:** Information criteria for multi-group latent class analysis models

**Model**	**-log likelihood**	**AIC**	**BIC**
Unconstrained	-3,390.623	6,879.246	7,105.367
Semiconstrained	-3,410.932	6,875.863	7,000.461
Semiconstrained, PCOS posterior probability variable	-3,404.259	**6,866.517**	**7,000.344**
Fully constrained	-3,420.879	6,893.758	7,013.74
Fully constrained, PCOS posterior probability variable	-3,417.608	6,891.216	7,020.428

The lowest values on the information criteria indicate the best model (shown in bold), weighing absolute model fit and number of estimated parameters. In the unconstrained model, both conditional probabilities and latent class prevalences are allowed to vary by diagnostic group; in the semi-constrained model, only latent class prevalences are allowed to vary by group; in the fully constrained model, both diagnositic groups are forced to have the same latent class prevalences and the same item-response conditional probabilities. Further discussion of model comparison can be found in Additional file 1, and 5-fold cross validated loglikelihood, AIC, and BIC values for these models can be found in Additional file 2. AIC, Akaike Information Criterion; BIC, Bayesian Information Criterion; *df*, degrees of freedom; PCOS, polycystic ovary syndrome.

**Table 4:** **Likelihood ratio tests used to compare model fit between H**_**0 **_**and H**_**1 **_**models**

**H**_**0 **_**Model**	**H**_**1 **_**Model**	**Δ *G *** **2**	***df***	***P *** **value**	**Null hypothesis (H**_**0**_**)**
Semiconstrained	Unconstrained	20.309	22	0.0625	Latent class characteristics equal between groups
Semiconstrained	Semiconstrained, PCOS posterior probability variable	6.673	2	**0.0178**	Constraining the PCOS parameter does not affect model fit
Fully constrained, PCOS posterior probability variable	Semiconstrained, PCOS posterior probability variable	13.349	1	**0.0001**	Latent class prevalences equal between groups

*P* value in bold indicates significance *P* < 0.05). *df*, degrees of freedom; PCOS, polycystic ovary syndrome.

When characteristics of the latent classes are similar between groups, we can restrain item-specific parameters to examine the prevalences of the latent classes between groups. However, because the PCOS parameter varied between groups, we allowed the groups to vary on this single parameter while holding the others constant (Figure 1). In order to test if the latent class prevalences differed between groups, we compared the semi-constrained model (only prevalences allowed to differ) to a fully-constrained model where latent class prevalences were forced to be the same for both the ASC and control groups. The semi-constrained model was a significantly better fit, indicating differences in latent class prevalence between groups (LRT; ΔG^2^ = 13.349, *df* = 1, *P* = 0.0001) (Table 4). We conclude that the primary difference between the ASC group and the control group was latent class prevalence: the model assigned 48% of individuals with ASC and 25% of controls to the ‘steroidopathic’ latent class (Table 5).

**Table 5:** Class prevalences and class-dependent conditional probabilities for conditions and symptoms relating to steroids

	**Latent class membership**	
	**Steroidopathic**	**Typical**
ASC	48%	52%
Control	25%	75%
**Posterior probabilities**	**Steroidopathic**	**Typical**
	**Conditional probability (95% CI)**	**Conditional probability (95% CI)**
Delayed puberty	0.01 (0.00-0.03)	0.01 (0.00-0.02)
Periods after 16 years	0.07 (0.04-0.11)	0.05 (0.03-0.06)
Periods before 10 years	0.12 (0.08-0.17)	0.03 (0.01-0.06)
Precocious puberty	0.05 (0.02-0.08)	0.00 (0.00-0.00)
Early growth spurt	0.26 (0.20-0.33)	0.10 (0.07-0.14)
PCOS (ASC/control)	0.24 (0.16-0.33)/0.47 (0.35-0.64)	0.00 (0.00-0.00)
Hirsutism	0.40 (0.32-0.48)	0.04 (0.02-0.07)
Irregular menstrual cycle	0.72 (0.63-0.80)	0.22 (0.18-0.27)
Painful periods	0.61 (0.54-0.68)	0.16 (0.11-0.21)
Acne	0.22 (0.17-0.28)	0.11 (0.08-0.15)
Excessive menstrual bleeding	0.56 (0.48-0.63)	0.10 (0.05-0.15)

Percentages of the ASC and control groups assigned to the steroidopathic and typical latent classes (top) and conditional probabilities for Testosterone-related Medical Questionnaire items by latent class (bottom). Bootstrapped (500 starts) 95% confidence intervals of parameter estimates in brackets. ASC, autism spectrum conditions; PCOS, polycystic ovary syndrome.

## Discussion

In line with previous results [45], we detected higher rates of items on the TMQ associated with atypical levels of, or atypical responses to, sex steroids among women with ASC. In particular, we confirmed higher frequencies of epilepsy, amenorrhea, dysmenorrhea, severe acne, gender dysphoria, and transsexualism, and differences in sexual preference in a large sample of women with ASC. Additionally, we detected significantly higher rates of anorexia, cardiac conditions, menorrhagia, PMS, precocious puberty, and early growth spurt among women with ASC. We did not replicate significant differences in PCOS diagnosis, delayed puberty, and hirsutism between women with ASC and controls.

In order to understand if equal rates of steroid-related conditions occurred between women with ASC and controls, we conducted a multi-group LCA on 11 items explicitly linked to sex steroids. In both women with ASC and controls, two latent classes (typical and steroidopathic) emerged; a higher percentage of women with ASC fell into the steroidopathic class than did controls. Increased membership of women with ASC in the steroidopathic class, which had high posterior probabilities for symptoms known to reflect circulating androgen levels, is consistent with elevated LH and bioavailable testosterone in women with ASC [7]. Within the steroidopathic groups, conditional probabilities on all items were equivalent between ASC/steroidopathic and control/steroidopathic, with the notable exception of PCOS. Because the steroidopathic groups were homogenous with respect to all other symptoms surveyed, we believe this is a diagnostic rather than a biological difference. It is possible that the reduced marriage rate and fecundity among women with ASC [51,52] make them less likely to have a diagnosis of PCOS as they may be less likely to have sought infertility treatment, contributing to possible under-detection of PCOS within this population. PCOS only accounts for 85% of cases of clinical hyperandrogenemia within the general population, and the different conditional probabilities for PCOS diagnosis between the two steroidopathic groups could also reflect an increase in difficult-to-detect hyperandrogenic conditions such as non-classical congenital adrenal hyperplasia among women with ASC [53].

### Puberty and prenatal androgen exposure

The increased sample size of this follow-up study allowed us to detect differences in the timing of puberty (‘precocious puberty’ and ‘early growth spurt’) between women with autism and controls. Adrenarche, or adrenal puberty, is marked by an increase in the adrenal androgens DHEA and androstenedione; elevation of adrenal androgens is mediated by birth weight, and catch-up growth [54,55], which responds to insulin-like growth factors [56,57]. Adrenarche occurs prematurely in low birth weight children, females who later develop PCOS, and daughters of females with PCOS [39], suggesting prenatal programming of adrenal androgen synthesis. Women with ASC reported precocious puberty more frequently than controls. Unfortunately, the questionnaire did not distinguish between central, or HPG-dependent precocious puberty, and pseudo-precocious puberty, typically due to adrenal androgen secretion [58]. We are currently collecting data on the timing of various adrenal (for example, pubic hair growth) and gonadal (for example, breast development) markers of puberty in this group.

Activation of the HPG axis occurs at gonadarche (in human Caucasian girls beginning around age 9 to 10 years), as increasing gonadotropin-releasing hormone (GnRH) pulse frequency and amplitude elevate circulating sex steroids, leading to physiological changes including menarche (which has a typical age of onset of 12 to 13 years in Caucasian girls). Previous studies report a delay in menarche among women with ASC [45,59]; however, the current study found an early growth spurt and accelerated menarche in women with ASC, consistent with findings of both early [55] and delayed [40] menarche in PCOS. Decreased 2D:4D ratio is associated with delayed menarche in the general population [60,61], but adiposity and low birth weight - which can both be induced by maternal hyperandrogenemia - tend to accelerate puberty, and this pattern is more frequently observed in clinical PCOS populations [62].

Because we allowed latent class membership to co-vary on BMI, it is unsurprising that the posterior probabilities for early growth spurt and periods before 10 years of age are increased in the steroidogenic class while the posterior probabilities for periods after 16 years of age and delayed puberty are relatively similar to the typical class, as we have incorporated adiposity in our model. In hyperinsulinemic hyperandrogenemia, androgens accelerate desensitization of GnRH neurons to negative feedback by progesterone, causing LH-producing high frequency GnRH pulses to occur earlier in life [63,64]. The balance between androgens and progesterone may be mediated by γ-aminobutyric acid (GABA)_A_ receptors on presynaptic GABAergic and postsynaptic GnRH neurons, as progesterone administration to ovariectomized females results in decreased GABAergic post-synaptic current, but dihydrotestosterone administration raises GABAergic post-synaptic current and frequency, eliminating the effects of progesterone and resulting in elevated LH [65,66]. PNA increases connectivity between GABAergic and GnRH neurons, and dysregulates AMPK-mediated glucose sensing in GnRH neurons, causing elevated GnRH pulse frequency and amplitude throughout the lifespan [67,68].

### Atypical sensitivities to sex steroids in autism spectrum conditions?

In addition to the reproductive symptoms analyzed in the LCA, we also uncovered significant differences in neurological and psychiatric states associated with sex steroids between women with ASC and controls. Seizure activity is exacerbated by menstrual cycle phase in approximately 40% of women with epilepsy [69]. As progesterone, estradiol, and the sex steroid metabolites allopregnanolone and androstenedione modulate neuronal excitation, fluctuations between pro- and anti-convulsant neurosteroids during the menstrual cycle contribute to epileptogenesis [70]. Consistent with the involvement of the sex steroids in catamenial epilepsy (a form of epilepsy that is sex hormone-sensitive), there is an increased prevalence of PCOS and other reproductive disorders among women with epilepsy [71]. However, the relationship between female reproductive disorders and epilepsy is complex, as both seizure activity [72] and valproate use [71] can disrupt the HPG axis. While the co-morbidity of epilepsy and autism is well established [73], few efforts have been made to explain the increased risk for epilepsy among females with autism [74] and the peak prevalence of epilepsy at adolescence [73] among individuals with ASC.

Neurosteroid modulation of GABA_A_ receptors offers an intriguing potential connection between autism risk, sex steroids, and epilepsy. An autism-like phenotype that includes seizures and social deficits can be established in mice through selective haplodeletion of the Na^+^_V_1.1 ion channel-encoding *SCN1A* gene in forebrain GABAergic interneurons [75]. Furthermore, this phenotype can be rescued with clonazepam, a positive allosteric modulator of the GABA_A_ receptor that shares a binding site with allopregnanolone [75,76]. As the most neurosteroid-sensitive GABA_A_ subunit, δ, is downregulated in response to chronic elevations of progesterone and/or allopregnanolone, changes in circulating neurosteroids during puberty [77] and during the menstrual cycle could increase risk of GABA_A_-dependent epilepsy among women and adolescents with ASC. Interestingly, allopregnanolone increases in response to both adrenocorticotropic hormone and GnRH [78], implicating both the adrenals and ovaries in its production; however, adrenocorticotropic hormone-stimulated allopregnanolone levels are decreased in women with PCOS [79], suggesting dysregulation of allopregnanolone in this population. The convergence between ASC, sex steroid metabolism, epilepsy, and GABA_A_ could be a target for future research, especially given findings that prenatal testosterone programs GABAergic signaling [80], particularly in sexually dimorphic areas that control reproductive function [67].

Similar to epilepsy, the increased prevalence of PMS (PMDD) and anorexia in women with ASC may represent shared psychiatric susceptibilities to hormones rather than shared alterations in sex steroids *per se.* Women with PMDD may have a severe experience of normal fluctuations in emotional processing and cognition across the menstrual cycle, and estrogen modification of serotonergic circuits and allopregnanolone modification of GABAergic signaling have both been implicated in the etiology of PMDD [81]. An increased prevalence of anorexia among women with ASC is consistent with suggestions that anorexia may have a similar cognitive phenotype to, and share risk factors with, ASC [82]. Individuals with anorexia score significantly higher than controls on the AQ [83,84], have similar deficits to individuals with ASC in executive function and central coherence [85], and first-degree family members of anorexic individuals report a higher number of DSM-IV pervasive developmental delay symptoms [86].

Women with ASC reported masculine sex-typical childhood behaviors (‘tomboyism’), gender dysphoria, and non-heterosexual orientations significantly more frequently than controls, consistent with the organizational effects of prenatal testosterone on sexually dimorphic brain structures, and sexual behaviors [87]. The increased frequency of cardiac conditions among women with ASC may reflect abnormalities in calcium channels that could affect both neurological and cardiac development, as seen in Timothy Syndrome [88].

## Conclusions

We find increased frequencies of conditions linked to atypical steroid metabolism and atypical psychological responses to steroids in females with ASC. Additionally, we find that women with ASC are significantly more likely to belong to a ‘steroidopathic’ group characterized by reproductive dysfunction, hyperandrogenism, and atypical pubertal development, despite being less likely to report a diagnosis of PCOS than controls. Given the shared hypothesis of prenatal androgen exposure for both ASC and PCOS, further elucidating the cognitive phenotype of women with PCOS and the reproductive phenotype of women with ASC could offer insight into both conditions.

## Abbreviations

11β-HSD-1: 11β-hydroxysteroid dehydrogenase type 1; AIC: Akaike Information Criterion; AQ: Autism Spectrum Quotient; ASC: autism spectrum conditions; BIC: Bayesian Information Criterion; BMI: body mass index; CAH: congenital adrenal hyperplasia; CP: contraceptive pill; FT: fetal testosterone; GABA: γ-aminobutyric acid; GnRH: gonadotropin-releasing hormone; HPG: hypothalamic-pituitary-gonadal; LCA: latent class analysisLH, luteinizing hormone; LMR: Lo-Mendell-Rubin; LRT: likelihood ratio test; PCOS: polycystic ovary syndrome; PMDD: premenstrual dysphoric disorder; PMS: premenstrual syndrome; PNA: prenatal androgenization; TMQ: Testosterone-related Medical Questionnaire.

## Competing interests

The authors declare that they have no competing interests.

## Authors’ contributions

AP: data collection and analysis, manuscript writing and final approval of the manuscript. SC: data collection, critical revision and final approval of the manuscript. BA: conception and design, critical revision and final approval of the manuscript. SB-C: conception and design, financial support, manuscript writing, final approval of the manuscript. All authors read and approved the final manuscript.

## Supplementary Material

Additional file 1 **Expanded Methods. **Expanded discussion of latent class modeling. **Expanded Results:** Expanded results of latent class model fit. **Table S1:** Posterior probabilites of two- and three-class models. **Appendix 1:** Testosterone-related Medical Questionnaire. **Figure S1:** Strong agreement on item posterior probabilities within groups and classes. Click here for file

Additional file 2 **Model Validation.** Results of five-fold cross validation. Click here for file
