# Supplementary material for: Uncovering steroidopathy in women with autism: a latent class analysis
Source: Mol Autism. 2014 Apr 9;5:27. doi: 10.1186/2040-2392-5-27 (PMC4022124; doi:10.1186/2040-2392-5-27)
Supplement: Additional file 1 — Expanded Methods. Expanded discussion of latent class modeling. Expanded Results: Expanded results of latent class model fit. Table S1: Posterior probabilites of two- and three-class models. Appendix 1: Testosterone-related Medical Questionnaire. Figure S1: Strong agreement on item posterior probabilities within groups and classes. [file 2040-2392-5-27-S1.docx]

**Supplementary Information 1**

**Expanded Methods: Latent Class Analysis**

Latent class analysis (LCA) assumes that an individual’s responses on a series of observed variables can be explained by an individual’s status on an unmeasured, or latent, categorical variable. LCA identifies high frequency response patterns on the series of observed variables, and these response patterns are used to draw conclusions about the discrete subpopulations, or latent classes, within the study population [1]. For each subpopulation identified, LCA returns item-specific conditional probabilities describing the likelihood that an individual would respond positively to specific items given membership in a given latent subpopulation. These conditional probabilities are used to characterize the subpopulations.

Latent class analysis is a form of finite mixture modeling, first described in detail by Lazarsfeld and Henry [2], and operationalized with the introduction of the expectation-maximization (EM) algorithm by Dempster, Laird and Rubin [3]. Latent class analysis uses the EM algorithm to determine 1) the latent classes within the population and 2) posterior probabilities for each item, conditioned on latent class membership.

Because latent class analysis hinges on accurate identification of the number of latent classes, we analyzed the ASC and control groups together in order to determine the number of latent classes, as recommended by Collins and Lanza [1]. The (Vuong)-Lo-Mendell-Rubin test was used to compare models with *k* and *k-1* latent classes, and the Akaike Information Criterion (AIC), and Bayesian Information Criterion (BIC) were used to compare model fit. As model fit improves as a consequence of increasing the number of latent classes, AIC and BIC penalize a model’s goodness-of-fit score by the number of estimated parameters. A better model will result in a lower AIC and BIC, which can be compared between models with different numbers of classes to determine relative model fit.

Multiple-group latent class analysis allows the detection of both qualitative differences (i.e. number of latent classes, class-dependent conditional probabilities that help us to describe and identify the classes) and quantitative differences (i.e. differences in the proportions of individuals in each class) between groups. Multigroup latent class models can be broken down into three types: unconstrained, where both posterior probabilities and latent class membership are allowed to vary between groups, semi-constrained, where posterior probabilities are held equivalent but latent class membership is allowed to vary, and fully constrained, where neither posterior probabilities nor latent class membership are allowed to vary between groups.

Comparing the unconstrained to the semi-constrained model tests whether the characteristics of the latent classes are similar between groups. In latent class analysis, equality between posterior probability sets for the same latent class given different group membership is referred to as *measurement invariance*. If measurement invariance holds for two groups, the characteristics of an individual given their latent class are the same regardless of group membership. For our data, measurement invariance would indicate that the likelihood of experiencing symptoms and/or conditions was consistent between women with ASC and controls. Under conditions of measurement invariance, differences in latent class prevalences between groups are assumed to reflect quantitative differences in the proportion of individuals from each group belonging to each latent class, rather than qualitative differences incurred by differential latent class assignment. Differences in latent class prevalences can be tested by comparing the semi-constrained to the fully constrained model, as the former allows class assignment to vary by group but the latter does not.

Likelihood ratio tests (LRTs) can be used to determine if a model with fewer constraints (H_1_) offers a significant improvement in fit over a nested model with fewer estimated parameters (H_0_) – the unconstrained, semi-constrained, and fully constrained models are all nested. The LRT test statistic, Δ*G*^2^, is the difference between the -2log likelihood (-2LL) of the more restrictive model (*G^2^*_0_) and the less restrictive model (*G^2^*_1_). Δ*G*^2^ has a Χ^2^ distribution, and its degrees of freedom are computed by subtracting the number of estimated parameters from the more parsimonious model (H_0_) from the number of estimated parameters of the more complex model (H_1_). The LRT can be used to test the null hypothesis of measurement invariance by comparing the unconstrained model to the semi-constrained model, and can also test the null hypothesis of no group differences in latent class prevalence by comparing the semi-constrained model to the fully-constrained model.

Finally, we performed a 5-fold cross validation procedure on the 5 multi-group latent class models in order to verify that the results of the LRTs corresponded to the model with the best performance in test replications (Supplementary Information 2). All latent class modeling was done with MPlus Version 7.

**Expanded Results: Latent Class Analysis**

As the number of latent classes must be specified by the researcher in LCA, we compared model fit for models with *k* classes using the AIC and BIC. Additionally, the (Vuong)-Lo-Mendell-Rubin (LMR) Likelihood test was computed to compare the model with *k* classes against the more parsimonious model with *k-1* classes. The 3-class model had the lowest AIC, but the 2-class model had the lowest BIC. The BIC tends to outperform the AIC at identifying the best model when the sample size is large in LCA [4]. The LMR test supported the 2-class model, as the 3-class model did not fit better than the 2-class model (p=0.6174). Finally, we examined posterior probabilities for item responses in the 2- and 3- class models (Supplementary Table 1). A cursory overview of the data suggested that the *k*=2 model identified one latent class with many steroid-related symptoms (population prevalence: 36%) and a latent class with far fewer symptoms (population prevalence: 64%). The *k*=3 model identified the same class lacking symptoms (population prevalence: 63%), but split individuals reporting symptoms into severely affected (population prevalence: 15%) and moderately affected (population prevalence: 21%) subgroups. Latent class prevalence can be estimated either by averaging posterior class membership probabilities of each set of observations, or by placing each set of observations in a latent class based on its single highest class posterior probability (modal classification). Strong agreement between classification methods indicates robust discrimination between latent classes in the model. Pairwise examination of posterior membership probabilities by modal classification in the *k*=2 and *k*=3 models suggested that the *k*=2 model provided the better fit for the data, as there was stronger agreement between the posterior probabilities and modal likelihoods in the *k*=2 model. Consequently, we determined that the 2-class model was the best representative of the true latent class structure, and used a *k*=2 model for our multigroup latent class analysis.

In order to ascertain whether latent class symptom characteristics and subpopulation structure varied by ASC diagnosis, we performed a 2-class mulitgroup LCA comparing diagnostic groups. First, we compared an unconstrained model (both latent class prevalence and item-response posterior probabilities allowed to differ by group) to a semi-constrained model (only latent class prevalence allowed to differ by group) in order to test whether the characteristics of the latent classes were the same between groups (measurement invariance). Though the characteristics of the classes were not significantly different between the ASC and control groups (ΔG^2^ =20.309, *df* =22, p = 0.0625) (Table 4, Supplementary Figure 1), the trend towards significance prompted us to further explore individual symptom posterior probabilities between diagnostic groups. Subsequently, a Wald parameter test revealed that the difference in the posterior probability of reporting PCOS was significant (Χ^2^=11.743, *df*=2, p=0.0028) between diagnostic groups; no other posterior probabilities differed significantly between groups. As the other class-dependent conditional probabilities showed good agreement between groups (Supplemental Figure 1), we elected to constrain all other symptom parameters but allow PCOS diagnosis probability to vary between groups. Allowing for partial measurement invariance by allowing posterior probabilities to vary for PCOS between groups improved the fit of our semi-constrained model on all information criteria (Table 3), and significantly improved model fit (LRT; ΔG^2^ = 6.673, *df* = 2, p = 0.0178), validating our decision. Finally, to determine whether latent class prevalences varied between groups, we compared the semi-constrained model with partial measurement invariance to a fully-constrained model with partial measurement invariance, and found that the semi-constrained model was a significantly better fit for our data (LRT; ΔG^2^ = 13.349, *df* = 1, p = 0.0001) (Table 4). Additionally, in a 5-fold cross validation procedure, the semi-constrained model with partial measurement invariance had the lowest information criterion values on 4 of 5 ‘test’ trials, confirming that it was the best-fitting model (Supplementary Information 2). From this semi-constrained model, we can conclude that the primary difference between the ASC group and the control group was latent class prevalences: the model assigned 48% of individuals with ASC and 25% of controls to the ‘steroidopathic’ latent class.

Supplementary Table 1: Posterior probabilities of 2- and 3- class models

|  | 2 Latent Class Model | | 3 Latent Class Model | | |
| --- | --- | --- | --- | --- | --- |
| Latent Class Prevalences | 36% | 64% | 15% | 21% | 63% |
|  | **Steroidopathic**  conditional probability (95% CI) | **Typical**  conditional probability (95% CI) | **Steroidopathic**  **(Severe)**  conditional probability (95% CI) | **Steroidopathic (Moderate)**  conditional probability (95% CI) | **Typical**  conditional probability (95% CI) |
| Delayed Puberty | 0.01  (0.00-0.03) | 0.01  (0.00-0.02) | 0.01  (0.00-0.05) | 0.00  (0.00-0.00) | 0.01  (0.00-0.02) |
| PCOS | 0.34  (0.26-0.46) | 0.00  (0.00-0.00) | 0.79  (0.42-1.00) | 0.03  (0.00-0.26) | 0.00  (0.00-0.00) |
| Precocious Puberty | 0.05  (0.02-0.08) | 0.00  (0.00-0.01) | 0.02  (0.00-0.06) | 0.06  (0.02-0.13) | 0.00  (0.00-0.00) |
| Hirsutism | 0.38  (0.32-0.51) | 0.05  (0.02-0.08) | 0.66  (0.45-0.80) | 0.17  (0.00-0.37) | 0.06  (0.02-0.09) |
| Irregular Menstrual Cycle | 0.69  (0.64-0.83) | 0.23  (0.18-0.27) | 0.88  (0.74-0.98) | 0.54  (0.24-0.71) | 0.24  (0.17-0.29) |
| Painful Periods | 0.61  (0.54-0.69) | 0.17  (0.11-0.22) | 0.54  (0.22-0.66) | 0.64  (0.42-093) | 0.14  (0.06-0.20) |
| Early Growth Spurt | 0.26  (0.20-0.33) | 0.11  (0.07-0.14) | 0.26  (0.12-0.35) | 0.24  (0.16-0.43) | 0.10  (0.05-0.14) |
| Periods After 16 | 0.07  (0.06-0.11) | 0.05  (0.03-0.07) | 0.10  (0.04-0.18) | 0.03  (0.00-0.08) | 0.05  (0.03-0.08) |
| Periods Before 10 | 0.12  (0.04-0.17) | 0.03  (0.01-0.06) | 0.11  (0.00-0.21) | 0.13  (0.05-0.30) | 0.03  (0.00-0.05) |
| Acne | 0.21  (0.16-0.27) | 0.12  (0.08-0.15) | 0.23  (0.12-0.32) | 0.19  (0.03-0.27) | 0.12  (0.09-0.016) |
| Excessive Menstrual Bleeding | 0.59  (0.50-0.66) | 0.08  (0.05-0.16) | 0.51  (0.10-0.64) | 0.64  (0.42-0.88) | 0.05  (0.01-0.11) |

Percentages of the ASC and control groups assigned to the steroidopathic and typical latent classes (top) and conditional probabilities for TMQ items by latent class (bottom). Bootstrapped (500 starts) 95% confidence intervals of parameter estimates in brackets.

Supplementary Appendix 1: Hormone Medical Questionnaire

| **1. Have you ever been diagnosed by a medical doctor with any of the following medical conditions?** |
| --- |
| *no diagnosis, PCOS, epilepsy, cardiac arrhythmia/atrial fibrillation, thyroid gland abnormalities, precocious puberty, penicillin allergy, ovarian cancer/tumors/growths, medical condition involving a hormonal treatment, breast cancer/tumors/growths, PMS, diabetes, anorexia, other cardiac condition, congenital adrenal hyperplasia (CAH), delayed puberty, uterine cancer/tumors/growths* |
| **2a. Have any of your close relatives (i.e. parents, siblings, grandparents, children) been diagnosed with any of the following?** |
| *breast cancer/tumors/growths, ovarian cancer/tumors/growths, uterine cancer/tumors/growths, prostate cancer* |
| **2b. If you have selected any diagnoses for close relatives in the question above, please specify which relative(s):** |
| **3. Have you had any of the following problems in adulthood?** |
| *excessive bodily or facial hair (hirsutism), unusually painful periods, one or more miscarriages, pre-eclampsia, irregular menstrual cycle, excessive menstrual bleeding or endometriosis, difficulty in conceiving* |
| **4. Did you have any of the following in the past?** |
| *periods began before the age of 10, severe acne, periods began after the age of 16, early growth spurt (e.g. being one of the tallest in your class at school)* |
| **5. Have you ever been pregnant or attempted to become pregnant?** |
| **6. Have you used the contraceptive pill?** |
| **7. How tall are you?** |
| **8. How much do you weigh?** |
| **9. Do you wear glasses / contact lenses to correct your vision?** |
| **10. Were you considered a tomboy as a child?** |
| **11. Have you ever been diagnosed with a gender identity disorder (GID)?** |
| **12. For which sex do you have a sexual preference?** |
| *male, female both, neither* |
| **13. Are you transsexual/transgendered?** |


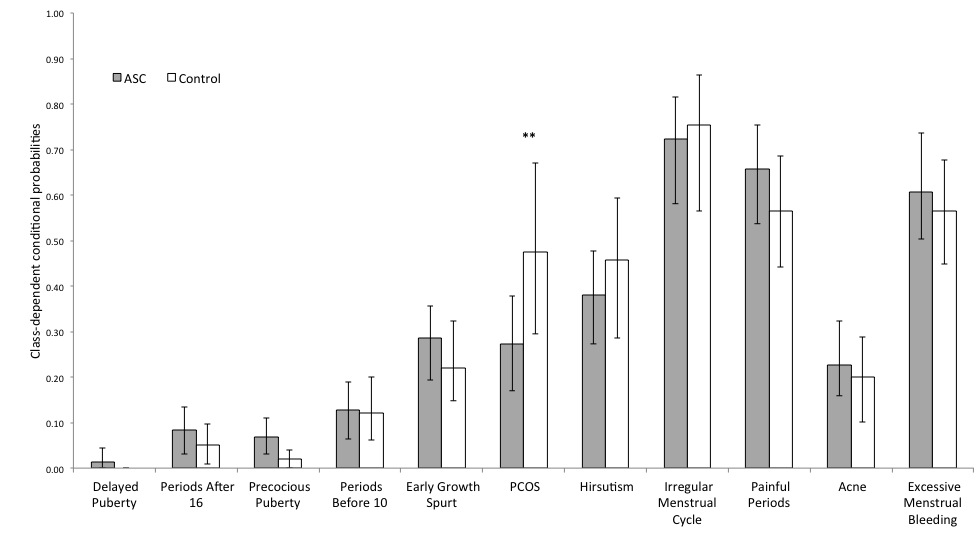


A

Steroidopathic


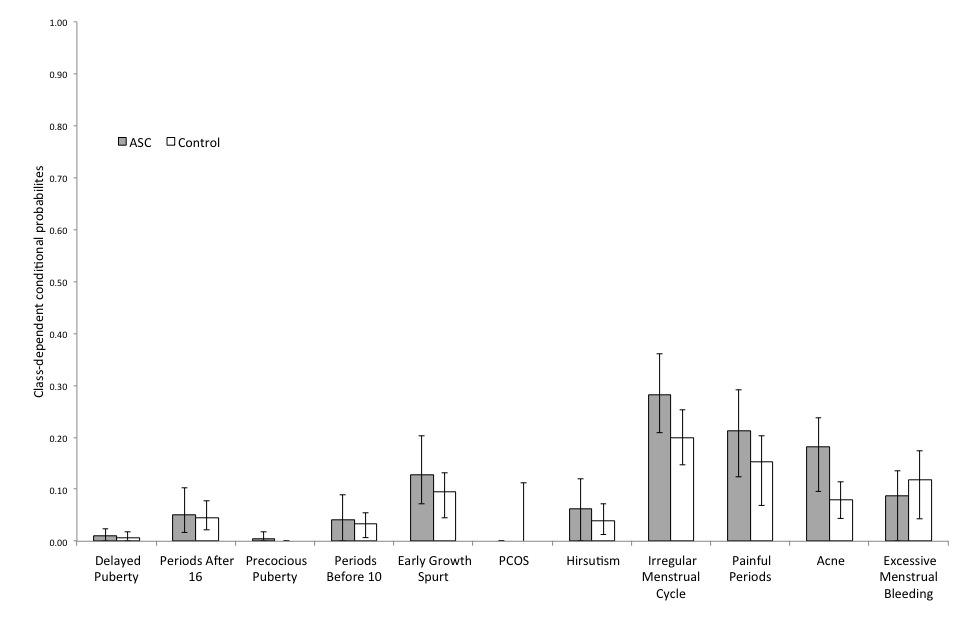


B

Typical

Supplemental Figure 1: Conditional probabilities for steroid-related conditions for the steroidopathic (A) and typical (B) latent classes by diagnosis indicating good agreement between groups for each latent class. This figure provides a visual representation of the concept of ‘measurement invariance’ between groups. Error bars represent bootstrapped (500 starts) 95% confidence intervals. Significant parameter differences between groups are marked with * (*p*<0.05 = *, *p*<0.01 = **).

1. Collins LM, Lanza ST: *Latent Class and Latent Transition Analysis: With Applications in the Social, Behavioral, and Health Sciences*. John Wiley & Sons; 2010.

2. Lazarsfeld PF, Henry NW: *Latent Structure Analysis*. Houghton, Mifflin; 1968.

3. Dempster AP, Laird NM, Rubin DB: **Maximum likelihood from incomplete data via the EM algorithm**. *J R Stat Soc* 1977, **39**:1–38.

4. Nylund K, Apsparouhov T, Muthen B: **Deciding on the number of classes in latent class analysis and growth mixture modeling: A Monte Carlo simulation study**. *Struct Equ Model A Multidiscip J* 2007, **14**:535–569.
