# Supplementary material for: Uncovering steroidopathy in women with autism: a latent class analysis
Source: Mol Autism. 2014 Apr 9;5:27. doi: 10.1186/2040-2392-5-27 (PMC4022124; doi:10.1186/2040-2392-5-27)
Supplement: Additional file 2 — Model Validation. Results of five-fold cross validation. [file 2040-2392-5-27-S2.docx]

**Supplemental Information 2**

**Model Validation**

In order to validate that we had selected the best-fitting model, we undertook a 5-fold cross validation procedure on the 5 multi-group models considered. The dataset was randomized and 5 folds of equal sizes were generated using MatLab. Four-fifths of the folds were used in the training dataset, and the last, unseen fold was used to evaluate the model (Collins & Lanza, 2010). We present a summary of the results of the last, ‘test’ folds below.

Table 1. Information criterion for 5-fold cross validation of multi-group latent class models

Unconstrained

| *k* fold | 1 | 2 | 3 | 4 | 5 |
| --- | --- | --- | --- | --- | --- |
| **loglikelihood** | -700.948 | **-651.605** | -721.338 | -670.659 | -706.789 |
| **AIC** | 1411.896 | **1313.210** | 1452.677 | 1351.318 | 1423.578 |
| **BIC** | 1427.049 | **1328.128** | 1467.763 | 1366.201 | 1438.664 |
| **aBIC** | 1411.223 | **1312.305** | 1451.938 | 1350.379 | 1422.840 |

Semi-constrained

| *k* fold | 1 | 2 | 3 | 4 | 5 |
| --- | --- | --- | --- | --- | --- |
| **loglikelihood** | -703.363 | -652.086 | -719.698 | -661.930 | -701.315 |
| **AIC** | 1416.725 | 1314.173 | 1449.397 | 1333.861 | 1412.629 |
| **BIC** | 1431.878 | 1329.091 | 1464.483 | 1348.744 | 1427.716 |
| **aBIC** | 1416.052 | 1313.268 | 1448.659 | 1332.923 | 1411.891 |

Semi-constrained, PCOS parameter variable

| *k* fold | 1 | 2 | 3 | 4 | 5 |
| --- | --- | --- | --- | --- | --- |
| **loglikelihood** | **-700.081** | -653.807 | **-718.126** | **-661.463** | **-700.677** |
| **AIC** | **1410.63** | 1317.614 | **1446.251** | **1332.926** | **1411.354** |
| **BIC** | **1425.315** | 1332.532 | 1461.338 | **1347.809** | **1426.441** |
| **aBIC** | **1409.490** | 1316.710 | **1445.513** | **1331.988** | **1410.616** |

Fully constrained

| *k* fold | 1 | 2 | 3 | 4 | 5 |
| --- | --- | --- | --- | --- | --- |
| **loglikelihood** | -705.363 | -654.447 | -719.989 | -663.114 | -703.201 |
| **AIC** | 1418.725 | 1316.894 | 1447.978 | 1334.227 | 1414.403 |
| **BIC** | 1430.847 | 1328.828 | 1460.047 | 1346.134 | 1426.472 |
| **aBIC** | 1418.187 | 1316.171 | 1447.388 | 1333.477 | 1413.812 |

Fully constrained, PCOS parameter variable

| *k* fold | 1 | 2 | 3 | 4 | 5 |
| --- | --- | --- | --- | --- | --- |
| **loglikelihood** | -703.531 | -656.196 | -719.855 | -666.411 | -705.586 |
| **AIC** | 1415.062 | 1320.391 | 1447.710 | 1340.822 | 1419.171 |
| **BIC** | 1427.183 | 1332.325 | **1459.779** | 1352.729 | 1431.241 |
| **aBIC** | 1414.523 | 1319.668 | 1447.119 | 1340.072 | 1418.581 |

AIC = Akaike Information Criterion, BIC = Bayesian Information Criterion, aBIC = sample-size adjusted BIC. The lowest information criterion values for a particular ‘test’ fold are highlighted in **bold.**

**References:**

Collins, L. M., & Lanza, S. T. (2010). *Latent Class and Latent Transition Analysis: With Applications in the Social, Behavioral, and Health Sciences*. John Wiley & Sons.
